# Identification of shared diagnostic biomarkers and immune-related mechanisms between sepsis and thoracic aortic aneurysm based on integrated bioinformatics and machine learning approaches

**DOI:** 10.1371/journal.pone.0359784

**Published:** 2026-10-08

**Authors:** Chengqun She, Hao Deng, Yaoguang Feng, Chaozhong Long, Juan Wu

**Affiliations:** Department of Cardiothoracic Surgery, The First Affiliated Hospital, Hengyang Medical School, University of South China, Hengyang City, Hunan Province, China; Army Medical University, CHINA

## Abstract

**Background:**

Sepsis and thoracic aortic aneurysm (TAA) share pathological features of inflammation and vascular dysfunction, yet their molecular connections remain unclear.

**Methods:**

Transcriptomic datasets of sepsis and TAA from GEO were integrated to identify shared differentially expressed genes (DEGs). Functional enrichment and gene set enrichment analyses were performed to explore potential pathways. Machine learning models were applied to identify candidate genes with discriminatory potential, followed by immune infiltration analysis and assessment of gene expression in lipopolysaccharide (LPS)-treated vascular smooth muscle cells (VSMCs).

**Results:**

A total of 193 shared DEGs were identified, mainly enriched in T cell activation, Th17 differentiation, and oxidative stress–related pathways. Five candidate genes (ARG1, IL18R1, LIX1L, TRIM32, and PRKCH) were selected through sequential machine-learning analysis and showed generally reproducible expression patterns across independent datasets. Their expression was associated with estimated immune cell proportions. In LPS-treated VSMCs, ARG1 and IL18R1 were upregulated, whereas LIX1L was downregulated.

**Conclusion:**

Sepsis and TAA share common transcriptional signatures involving immune dysregulation and metabolic stress. ARG1 and IL18R1 may represent candidate molecular features associated with inflammatory and vascular responses.

## Introduction

Sepsis is a life-threatening syndrome characterized by dysregulated host immune responses to infection, leading to systemic inflammation, endothelial injury, and multiple organ dysfunction [1]. During sepsis, sustained activation of immune and endothelial cells triggers excessive cytokine release, microvascular thrombosis, and oxidative stress, which not only damage peripheral organs but also exert long-term effects on the vascular system [2]. Recent evidence indicates that even after apparent clinical recovery, patients with sepsis may experience persistent vascular dysfunction, increased arterial stiffness, and abnormal remodeling of large vessels [3]. These alterations suggest that the inflammatory and immune consequences of sepsis may be associated with chronic vascular diseases, including aortic aneurysm formation [4].

Thoracic aortic aneurysm (TAA) is a progressive degenerative disorder characterized by pathological dilation and weakening of the aortic wall [5]. While genetic factors such as mutations in fibrillin-1 and smooth muscle contractile proteins contribute to hereditary forms of TAA, increasing attention has been directed toward the role of inflammation and immune-mediated extracellular matrix degradation in sporadic cases [6]. Activated macrophages and T lymphocytes infiltrate the aortic wall, releasing pro-inflammatory cytokines and matrix metalloproteinases that accelerate elastin fragmentation and smooth muscle apoptosis [6,7]. Endothelial dysfunction further amplifies oxidative stress and inflammatory signaling, creating a vicious cycle of vascular injury and remodeling. Thus, chronic immune activation is increasingly recognized as a major contributor to TAA pathogenesis, linking systemic inflammatory stimuli to localized vascular degeneration [8].

Given these overlapping pathological processes, sepsis and TAA may share common molecular pathways associated with immune dysregulation, oxidative stress, and extracellular matrix remodeling [9,10]. However, the shared transcriptomic signatures and key molecular regulators connecting acute systemic inflammation and chronic vascular degeneration remain poorly defined [11]. Clarifying these molecular intersections would provide insight into how post-septic inflammation transitions to vascular structural damage, and potentially identify biomarkers predictive of aortic complications in patients recovering from sepsis. TAA was selected as the representative vascular disease because it is a prototypic large vessel degenerative disorder with strong evidence of immune mediated pathogenesis. In contrast, the links between sepsis and more common vascular outcomes such as atherosclerosis or peripheral artery disease have been relatively better characterized. The relationship between acute systemic inflammation and TAA remains underexplored, warranting this investigation.

In this study, we conducted an integrated transcriptomic and computational analysis to explore the shared molecular landscape between sepsis and thoracic aortic aneurysm. By combining multi-cohort gene expression profiling with machine learning-based feature selection, we sought to identify candidate genes and immune-related pathways shared between the two diseases.

## Methods

### Microarray data collection and processing

The two original expression profile datasets for Sepsis and control groups, including GSE95233 [12] and GSE57065 [13], were downloaded from the GEO database (https://www.ncbi.nlm.nih.gov/geo/). GSE95233 includes 51 sepsis patients and 22 healthy controls. The data were generated using the Affymetrix Human Genome U133 Plus 2.0 Array (Platform ID: GPL570), a gene expression microarray technology manufactured by Affymetrix, Inc. (Santa Clara, CA, USA). Similarly, GSE57065 includes 28 sepsis patients and 25 healthy controls, utilizing the same platform (GPL570). The datasets for TAA and control groups were GSE9106 [14] and GSE57691 [15]. GSE9106 includes 58 TAA patients and 36 healthy controls. The data were generated using the ABI Human Genome Survey Microarray Version 2 (Platform ID: GPL2986), a spotted oligonucleotide microarray manufactured by Applied Biosystems (Rockville, MD, USA). GSE57691 includes 49 TAA patients and 10 healthy controls. The data were generated using the Illumina HumanHT-12 V4.0 Expression BeadChip (Platform ID: GPL10558), an oligonucleotide bead-based microarray manufactured by Illumina Inc. (San Diego, CA, USA). To ensure transparency and reproducibility, the detailed characteristics of all included transcriptomic datasets are summarized in Table 1.

**Table 1:** Characteristics of the included transcriptomic datasets.

Dataset ID	GSE95233	GSE57065	GSE9106	GSE57691
Disease	Sepsis	Sepsis	TAA	AAA
Platform ID	GPL570	GPL570	GPL2986	GPL10558
Platform Name	Affymetrix Human Genome U133 Plus 2.0 Array	Affymetrix Human Genome U133 Plus 2.0 Array	ABI Human Genome Survey Microarray Version 2	Illumina HumanHT-12 V4.0 Expression BeadChip
Technology Type	Gene expression microarray	Gene expression microarray	Spotted oligonucleotide microarray	Oligonucleotide bead-based microarray
Species	Homo sapiens	Homo sapiens	Homo sapiens	Homo sapiens
Sample Source	Whole blood	Whole blood	Peripheral blood	Aortic tissue
Case (n)	51	28	58	49
Control (n)	22	25	36	10
Age range	25-85	19-84	Unknown	Unknown
Gender	F:30/M:43	F:29/M:24	Unknown	Unknown
Co morbidity disease	Unknown	Unknown	Unknown	Unknown
Sepsis Etiology	Bacterial sepsis	Bacterial sepsis	/	/

Note: F: Female; M: Male.

### Differential expression gene analysis

The study workflow is illustrated in Fig 1. DEG analysis was performed using the “Limma” package [16] in R software (version 4.3.1) for the Sepsis dataset (GSE95233) and TAA dataset (GSE9106). The cut-off criteria for DEGs were set as adj.P.Val < 0.05 and |log(FC)| > 0.5. Subsequently, the expression patterns of DEGs were visualized using the “pheatmap” package and the “ggplot2” package in R software. Heatmaps and volcano plots were generated to represent the expression patterns of DEGs. Low expression genes were not pre filtered, as the stringent thresholds together with subsequent machine learning screening were applied to ensure robustness. Each dataset was normalized separately. Probe IDs were converted to gene symbols using the corresponding platform annotation files. Because the included datasets were generated from different microarray platforms and biological compartments, direct merging was avoided to minimize potential platform-related and tissue-specific biases. Differential expression analysis was performed individually for each dataset. Shared DEGs were identified by taking the intersection of DEGs from each dataset rather than from a merged dataset (Step 1 of Fig 1).

**Fig 1:**
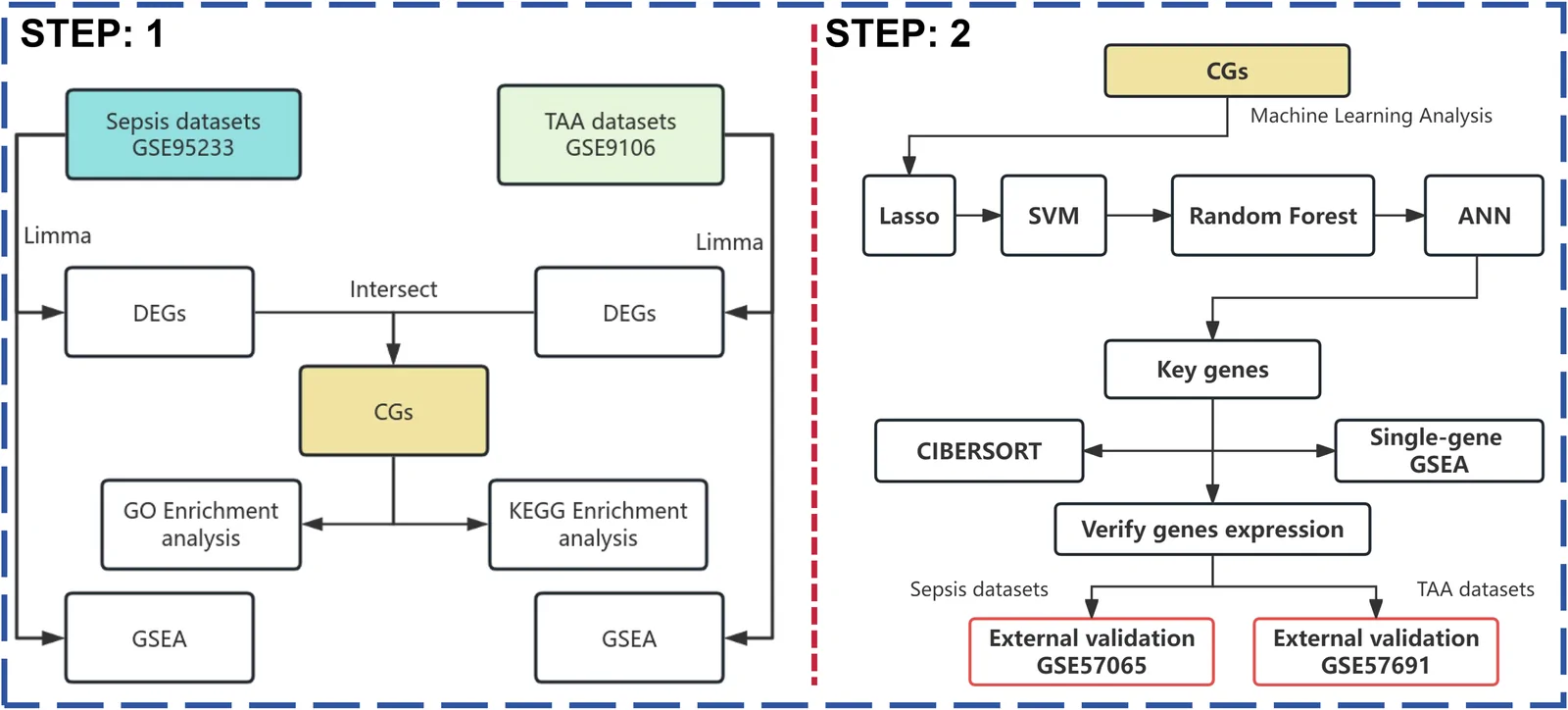
Flowchart of the study design.

### Function enrichment analysis

To explore the biological functions and specific mechanisms of the disease-related genes in TAA and Sepsis, we conducted GO and KEGG pathway enrichment analysis on the input gene set using packages such as “org.Hs.e.g.,db” [17], “GOplot” [18], “enrichplot” [19], and “clusterProfiler” [19]. A significance threshold of *P* < 0.05 was used to determine significant enrichment. To visualize the enrichment analysis results, we utilized packages such as “ggplot2,” “circlize,” “RColorBrewer,” and “ComplexHeatmap.” The results of the functional enrichment analysis were presented using circular plots and bubble plots.

### Gene set enrichment analysis

To complement the enrichment analysis based on shared differentially expressed genes and to characterize pathway-level alterations across the broader transcriptomic profiles, gene set enrichment analysis (GSEA) was performed separately in the sepsis dataset GSE95233 and the TAA dataset GSE9106. Genes in each dataset were ranked according to their differential expression between the disease and control groups. GSEA was performed using the R packages “clusterProfiler” and “org.Hs.e.g.,db.” The “c2.cp.kegg.Hs.symbols.gmt” was used as a reference gene set. The permutation number was set to 10,000, and the significance threshold was set at p < 0.05. Finally, the results were visualized using the R package “enrichplot.”

### Machine learning

To identify candidate genes with discriminatory potential shared between sepsis and TAA, we applied a sequential machine-learning framework using the shared DEGs identified in both conditions. The sepsis dataset was used for model development because it provided a clearly defined disease-control comparison and was suitable for identifying genes associated with inflammatory states. We hypothesized that genes consistently dysregulated in both sepsis and TAA and capable of discriminating sepsis from controls may represent shared inflammation-associated molecular features between the two conditions. The sepsis dataset used for model development was randomly split into training (70%) and testing (30%) sets, with five-fold cross-validation applied during model training. Lasso regression was initially used for feature selection [20,21], followed by SVM to assess the classification performance of the selected features [22]. The retained features were subsequently evaluated using random forest (RF), which ranked their relative importance based on MeanDecreaseGini [23]. A threshold of 4, determined by a natural break in the importance distribution, was used for further feature selection. The resulting candidate genes were then evaluated using an artificial neural network (ANN) as a complementary classification approach [24]. This sequential strategy was used to progressively refine the candidate gene set and assess the classification performance of the selected features (Fig 1, Step 2) [25,26].

### Validation of the expression levels of selected genes in sepsis and TAA

To assess the reproducibility of the expression patterns of the five selected genes across independent cohorts, we performed expression validation in both sepsis and TAA datasets. The genes were evaluated in the original datasets (GSE95233 for sepsis and GSE9106 for TAA) and subsequently validated in the independent external datasets (GSE57065 for sepsis and GSE57691 for TAA).

### Immune infiltration analysis

To investigate whether the shared molecular signatures between sepsis and TAA were associated with alterations in the immune cell composition, immune cell infiltration was estimated in the GSE95233 sepsis dataset and GSE9106 TAA dataset using the CIBERSORT algorithm [27]. CIBERSORT with the LM22 signature matrix was used to estimate the relative proportions of 22 immune cell types in each sample. Differences in the estimated proportions of individual immune cell types between the disease and control groups were assessed using the Wilcoxon rank-sum test. The distributions of immune cell proportions across samples were visualized using the “ggplot2” package. Correlations among the estimated proportions of the 22 immune cell types were assessed and visualized using the “corrplot” package. A *P* value < 0.05 was considered statistically significant.

### Single gene GSEA

To explore the biological pathways potentially associated with ARG1 and LIX1L, which showed consistent differential expression in the transcriptomic datasets and were further supported by the in vitro validation, single-gene GSEA was performed separately in the sepsis dataset GSE95233 and the TAA dataset GSE9106. For each gene, samples were stratified according to its expression level, and pathway enrichment patterns associated with gene expression were evaluated. Gene annotation and visualization were performed using the R packages “org.Hs.e.g.,db”, “ggsci”, “patchwork”, and “ggplot2”.

### Expression assessment of selected genes in LPS-treated VSMCs

To experimentally assess whether the expression patterns of the five selected genes could be reproduced under inflammatory conditions in vascular smooth muscle cells, their mRNA expression levels were evaluated in LPS-treated A7R5 cells. The A7R5 rat aortic smooth muscle cell line was cultured in DMEM supplemented with 10% fetal bovine serum (FBS). At approximately 80% confluence, cells were treated with lipopolysaccharide (LPS, 1 μg/mL) for 24 h to induce an inflammatory response. Total RNA was isolated using TRIzol reagent and reverse-transcribed into cDNA. The mRNA expression levels of the five selected genes were quantified by quantitative real-time PCR (qRT-PCR) using SYBR Green, with GAPDH as the internal control. Relative gene expression was calculated using the 2^ − ΔΔCt method.

Arg1 F: GGATAAACAGATCCAACCCGAT
R: TCCTGAAAGTAGCCCTGTCTTG
IL18R1 F: TGGGTTGCCTGATCCCTCTA
R: GCGAATGCAACTTTTCGGGG
LIX1L:F:GGGCTATGGTCGAGTGAACG
R:AATCTTTGCAGCACTTCGCC
PRKCH:F:CAGCGCCAGCATGTCGTC
R: CTCTCTGGAGAGTGGCTTCAGT
TRIM32:F:GGGCGGTCAGCAGGAAT
R: TGAAGAACTTCCGAGAGCGG
GAPDH:F:TGAAGCAGGCATCTGAGGG
R:CGAAGGTGGAAGAGTGGGAG

### Statistical analysis

GraphPad Prism version 9.0.2 (GraphPad Software Inc., San Diego, CA, USA) was used for statistical analysis. Results were displayed as mean  ±  SD. Differences between the two groups were compared by unpaired Student′s t-test. *P*  <  0.05 was regarded as statistical significance.

## Results

### Sepsis and TAA share 193 differentially expressed genes

To characterize transcriptional alterations in sepsis and TAA and identify genes dysregulated in both conditions, differential expression analyses were performed separately in the two datasets. In the sepsis dataset, 4,418 DEGs were identified between sepsis and control samples, comprising 2,330 upregulated and 2,088 downregulated genes. Their expression patterns were visualized using a volcano plot and heatmap (Fig 2A, B). In the TAA dataset, 1,322 DEGs were identified between TAA and control samples, comprising 314 upregulated and 1,018 downregulated genes (Fig 2C, D). Venn diagram analysis using the VennDiagram package [28] identified 193 DEGs shared between sepsis and TAA (Fig 2E). The complete list of the 193 shared DEGs is provided in Supplementary S1 Table.

**Fig 2:**
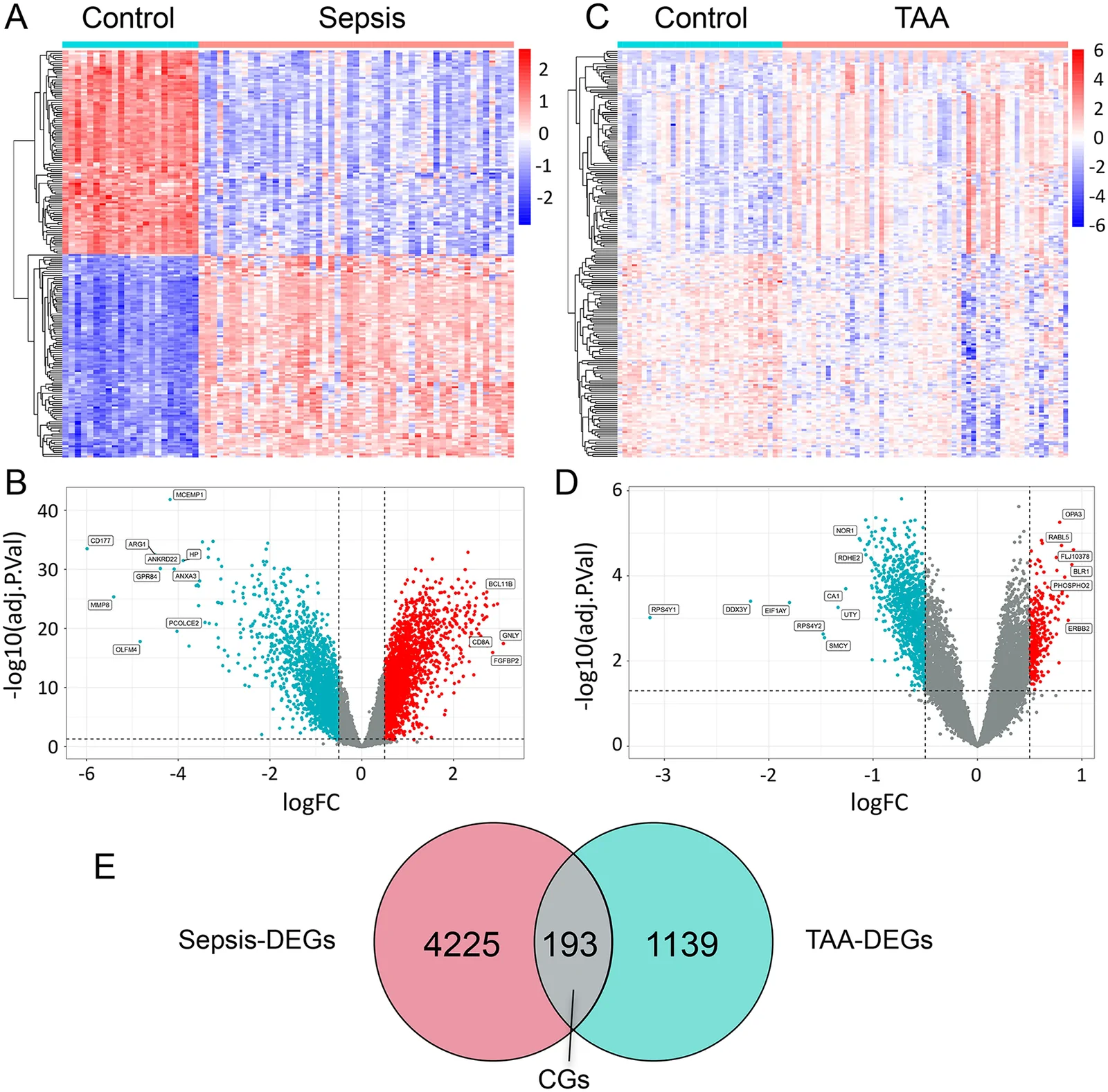
Differentially expressed gene analysis of the sepsis and TAA datasets. A, C: Heatmaps of the top 100 upregulated and 100 downregulated DEGs (adjusted *P* < 0.05, |logFC| > 0.5) in the sepsis and TAA datasets, respectively. B, D: Volcano plots showing DEGs in the sepsis and TAA datasets, respectively, with green indicating downregulation and red indicating upregulation. E: Venn diagram showing the intersection of DEGs between the sepsis and TAA datasets, identifying 193 shared DEGs.

### Shared transcriptional alterations highlight immune-related biological processes and pathways

To characterize the biological functions and pathways associated with the 193 shared DEGs, Gene Ontology and KEGG pathway enrichment analyses were performed. Gene Ontology analysis showed that the shared DEGs were mainly enriched in biological processes including “regulation of T cell activation,” “B cell activation,” “immune system development,” and “regulation of leukocyte apoptotic process.” Cellular component analysis indicated enrichment in the “specific granule lumen,” “specific granule,” and “endocytic vesicle lumen,” whereas molecular function analysis identified “immune receptor activity” as a prominent enriched term (Fig 3A). KEGG pathway analysis further showed enrichment in pathways including “primary immunodeficiency,” “Th17 cell differentiation,” “T cell receptor signaling pathway,” and “acute myeloid leukemia” (Fig 3B).

**Fig 3:**
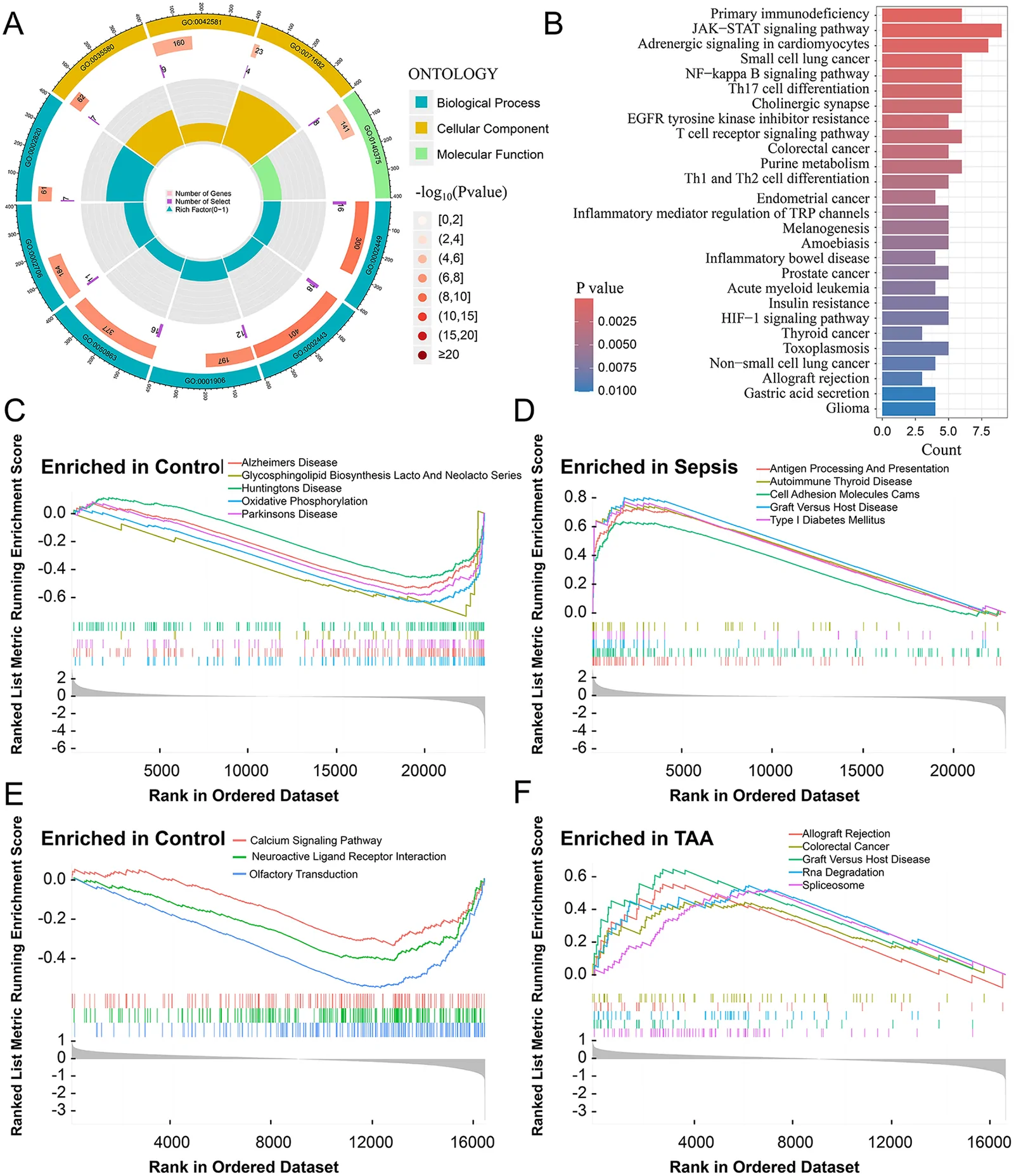
Functional enrichment and GSEA of shared transcriptional alterations. A: Circular plot illustrating the results of GO enrichment analysis for the 193 shared DEGs (p < 0.05). B: Bar chart representing KEGG pathway enrichment analysis for the 193 shared DEGs (p < 0.05). C, D: GSEA of the sepsis dataset (GSE95233), highlighting significant pathways (FDR < 0.05). E, F: GSEA of the TAA dataset (GSE9106), identifying key enriched gene sets (FDR < 0.05).

To complement the enrichment analysis of the shared DEGs and characterize pathway-level alterations across the broader transcriptomic profiles, GSEA was performed separately in the sepsis and TAA datasets. In the sepsis dataset, GSEA identified enrichment of “oxidative phosphorylation” (NES = 1.87, FDR = 0.026), “antigen processing and presentation” (NES = 1.65, FDR = 0.041), and “cell adhesion molecules” (NES = 1.58, FDR = 0.038) (Fig 3C, D). In the TAA dataset, enriched pathways included “calcium signaling pathway” (NES = 1.72, FDR = 0.031), “neuroactive ligand-receptor interaction” (NES = 1.59, FDR = 0.044), and “allograft rejection” (NES = 1.63, FDR = 0.036) (Fig 3E, F).

### Machine learning identifies five candidate genes shared between sepsis and TAA

To identify candidate genes with discriminatory potential, the 193 shared DEGs were sequentially evaluated using Lasso regression, svm, rf, and ann. Lasso regression initially reduced the candidate set to 14 genes (Fig 4A, B). These genes were subsequently evaluated by svm, followed by rf-based variable importance analysis. Based on the rf importance ranking, the top five ranked genes were LIX1L, PRKCH, TRIM32, ARG1, and IL18R1 (Fig 4C, D). These five candidate genes were further evaluated using ann, in which LIX1L and ARG1 showed importance scores exceeding 20 (Fig 4E, F). The classification performance of the selected features was further assessed across the machine learning models. Lasso regression yielded a mean cross-validated AUC of 0.892. The svm classifier achieved an accuracy of 0.857, with a sensitivity of 0.833 and a specificity of 0.875. Rf yielded an AUC of 0.914. ann achieved an accuracy of 0.886, a sensitivity of 0.867, a specificity of 0.900, and an AUC of 0.925.

**Fig 4:**
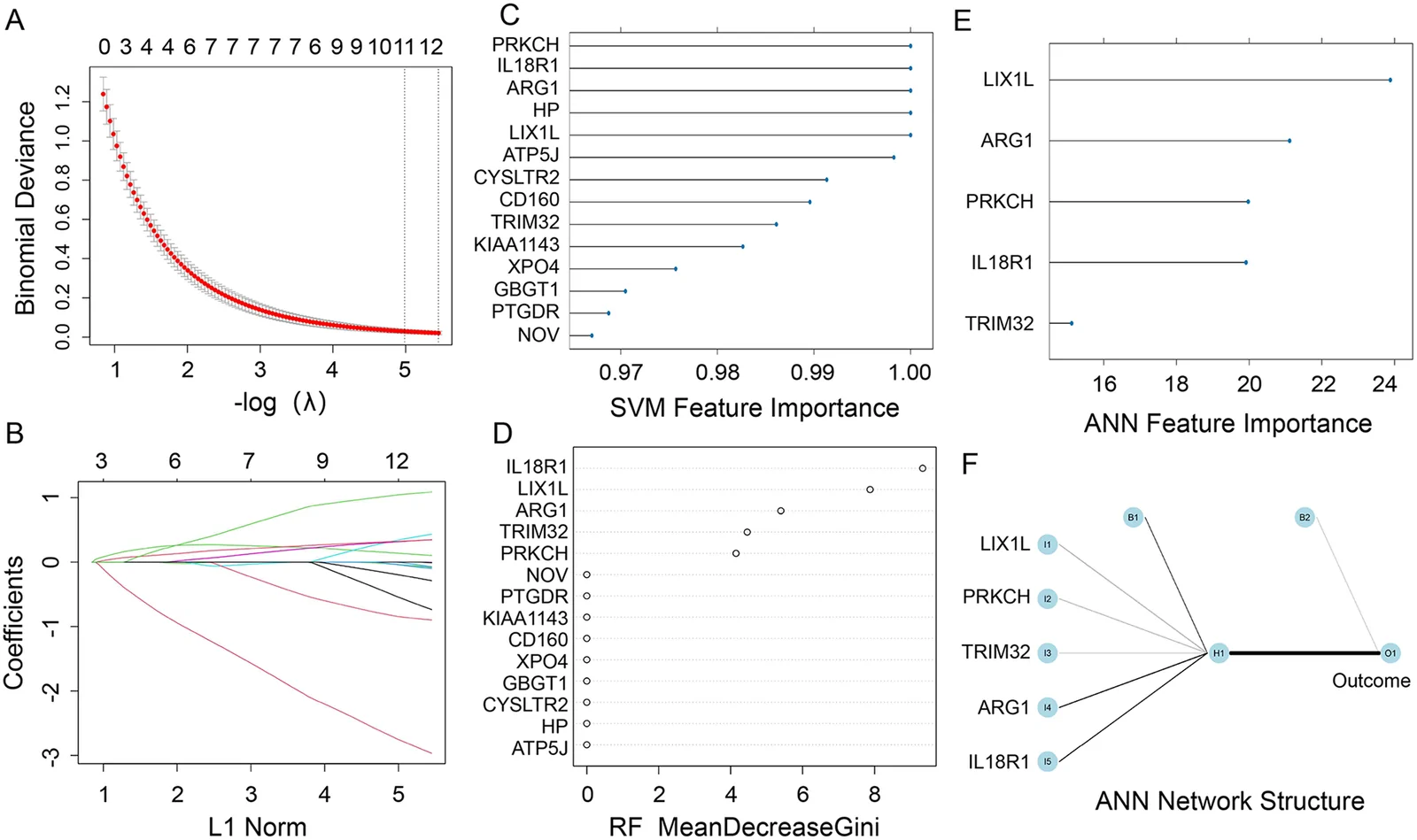
Sequential machine-learning analysis of candidate genes shared between sepsis and TAA. A, B: Lasso regression was applied to the 193 shared DEGs for initial feature selection, retaining 14 candidate genes. C: Classification performance of the Lasso-selected genes was assessed using SVM. D: RF-based variable importance ranking of the retained features. E, F: The five top-ranked candidate genes were further evaluated using ANN analysis.

### Selected genes show reproducible expression patterns across sepsis and TAA datasets

To assess the reproducibility of the five genes selected through the machine learning analysis, their expression patterns were examined in the original and independent external datasets for sepsis and TAA. In the original sepsis dataset GSE95233, ARG1 and IL18R1 were significantly upregulated in sepsis compared with controls, whereas LIX1L, TRIM32, and PRKCH were significantly downregulated (all *P* < 0.05; Fig 5C). Consistent expression patterns were observed for all five genes in the independent sepsis dataset GSE57065 (Fig 5D).

**Fig 5:**
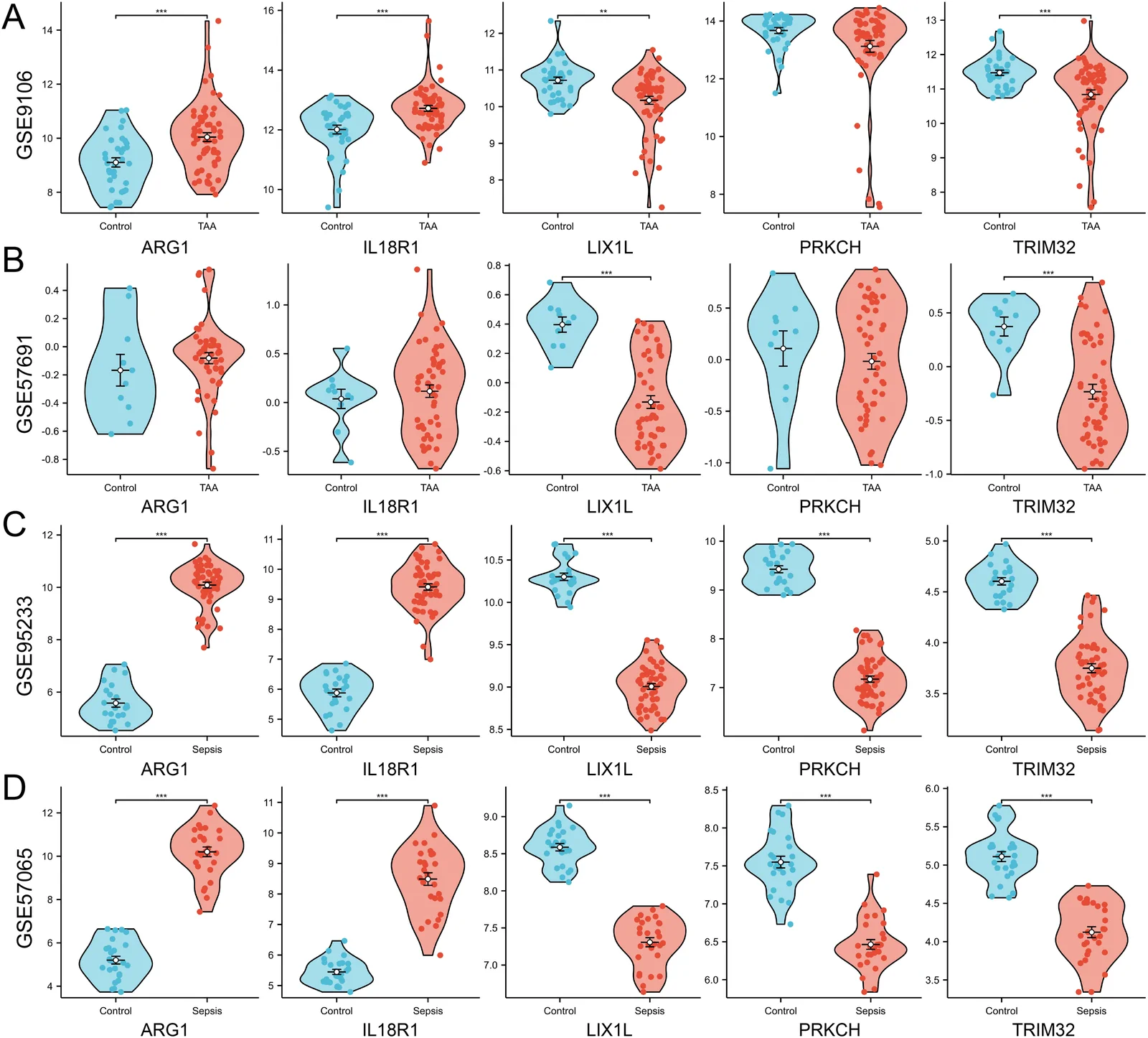
Expression patterns of the five selected genes across independent datasets. A, B: Expression levels of the five selected genes in the TAA dataset GSE9106 and the independent aortic aneurysm dataset GSE57691, respectively. C, D: Expression levels of the five selected genes in the sepsis datasets GSE95233 and GSE57065, respectively.

In the original TAA dataset GSE9106, ARG1 and IL18R1 were significantly upregulated, whereas LIX1L and TRIM32 were significantly downregulated in TAA compared with controls (all *P* < 0.05; Fig 5A). PRKCH showed a downward trend, although the difference did not reach statistical significance (*P* > 0.05). The expression patterns of the five selected genes in the independent TAA dataset GSE57691 were consistent with those observed in GSE9106 (Fig 5B). Overall, the five selected genes showed generally reproducible expression patterns across the original and independent datasets for both sepsis and TAA.

### ARG1, IL18R1, and LIX1L show consistent expression changes in LPS-treated VSMCs

To determine whether the expression patterns of the five selected genes could be reproduced under inflammatory conditions in vascular smooth muscle cells, their mRNA expression levels were examined in LPS-treated VSMCs. ARG1 and IL18R1 were significantly upregulated in LPS-treated VSMCs compared with control cells, whereas LIX1L was significantly downregulated (*P* < 0.05). In contrast, no significant differences in PRKCH or TRIM32 expression were observed between LPS-treated and control cells (*P* > 0.05) (Fig 6A).

**Fig 6:**
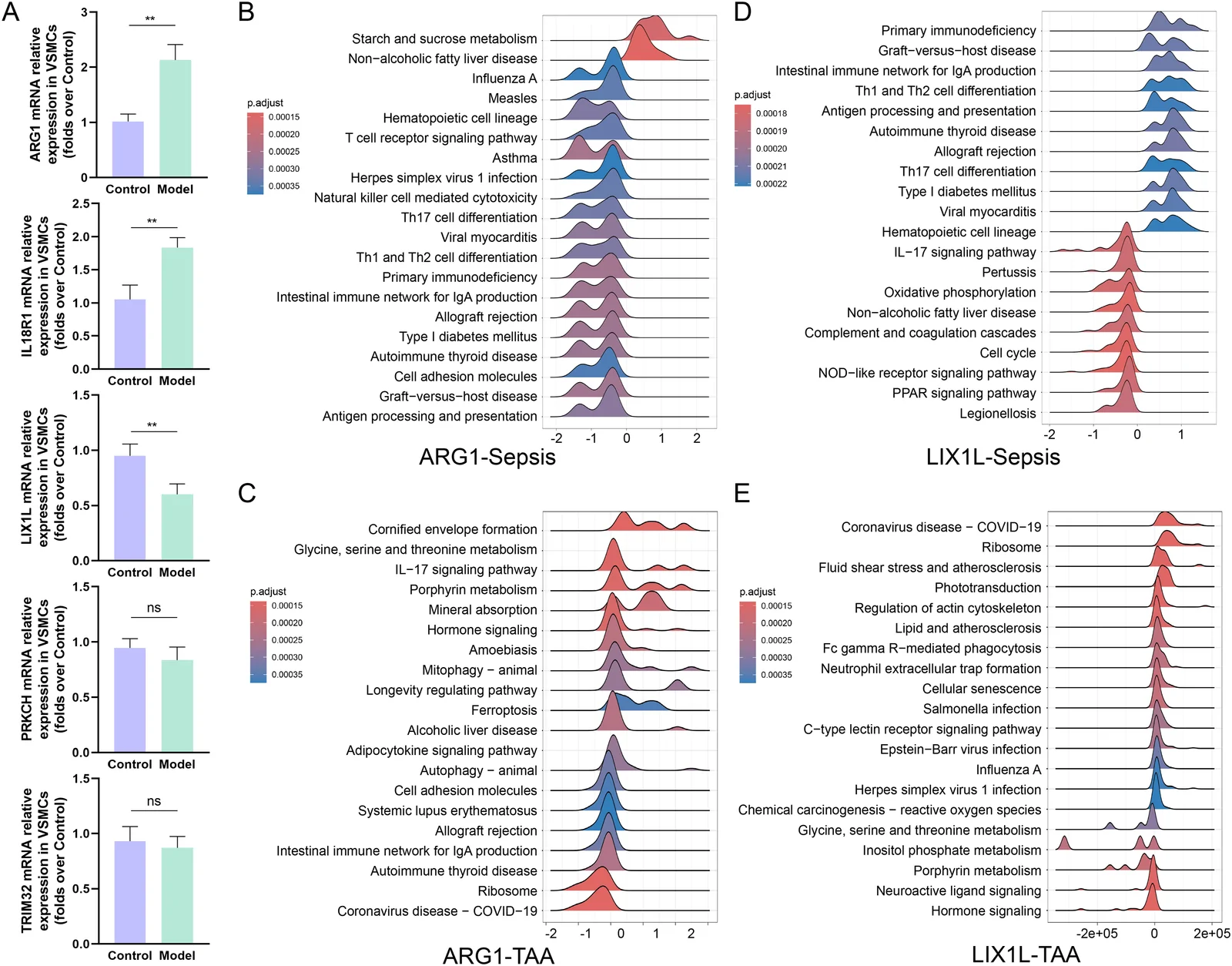
Expression assessment in LPS-treated VSMCs and single-gene GSEA. A: mRNA expression levels of the five selected genes in LPS-treated VSMCs. B: GSEA results of ARG1 in the sepsis dataset GSE95233. C: GSEA results of ARG1 in the TAA dataset GSE9106. D: GSEA results of LIX1L in the sepsis dataset GSE95233. E: GSEA results of LIX1L in the TAA dataset GSE9106.

### ARG1 and LIX1L are associated with distinct pathway enrichment patterns

To further characterize the biological pathways associated with ARG1 and LIX1L expression, single-gene GSEA was performed in the sepsis and TAA datasets. ARG1 expression was associated with 83 enriched pathways, including “starch and sucrose metabolism,” “non-alcoholic fatty liver disease,” “antigen processing and presentation,” “IL-17 signaling pathway,” “PPAR signaling pathway,” and “cell cycle” (Fig 6B, C). LIX1L expression was associated with 101 enriched pathways, including “IL-17 signaling pathway,” “ferroptosis,” “cellular senescence,” “hormone signaling,” “chemical carcinogenesis – reactive oxygen species,” and “autoimmune thyroid disease” (Fig 6D, E).

In the GSE95233 dataset, 14 of the 22 immune cell types estimated by CIBERSORT differed significantly between the sepsis and control groups (Fig 7A, B). Compared with controls, the sepsis group showed higher proportions of gamma delta T cells, monocytes, neutrophils, M0 macrophages, and eosinophils, and lower proportions of naive B cells, CD8 T cells, naive CD4 T cells, resting mast cells, and resting NK cells. The immune score was significantly lower in the sepsis group than in the control group (Fig 7C). Correlation analysis further showed significant associations between the five selected genes, LIX1L, PRKCH, TRIM32, ARG1, and IL18R1, and immune cell proportions (Fig 7D).

**Fig 7:**
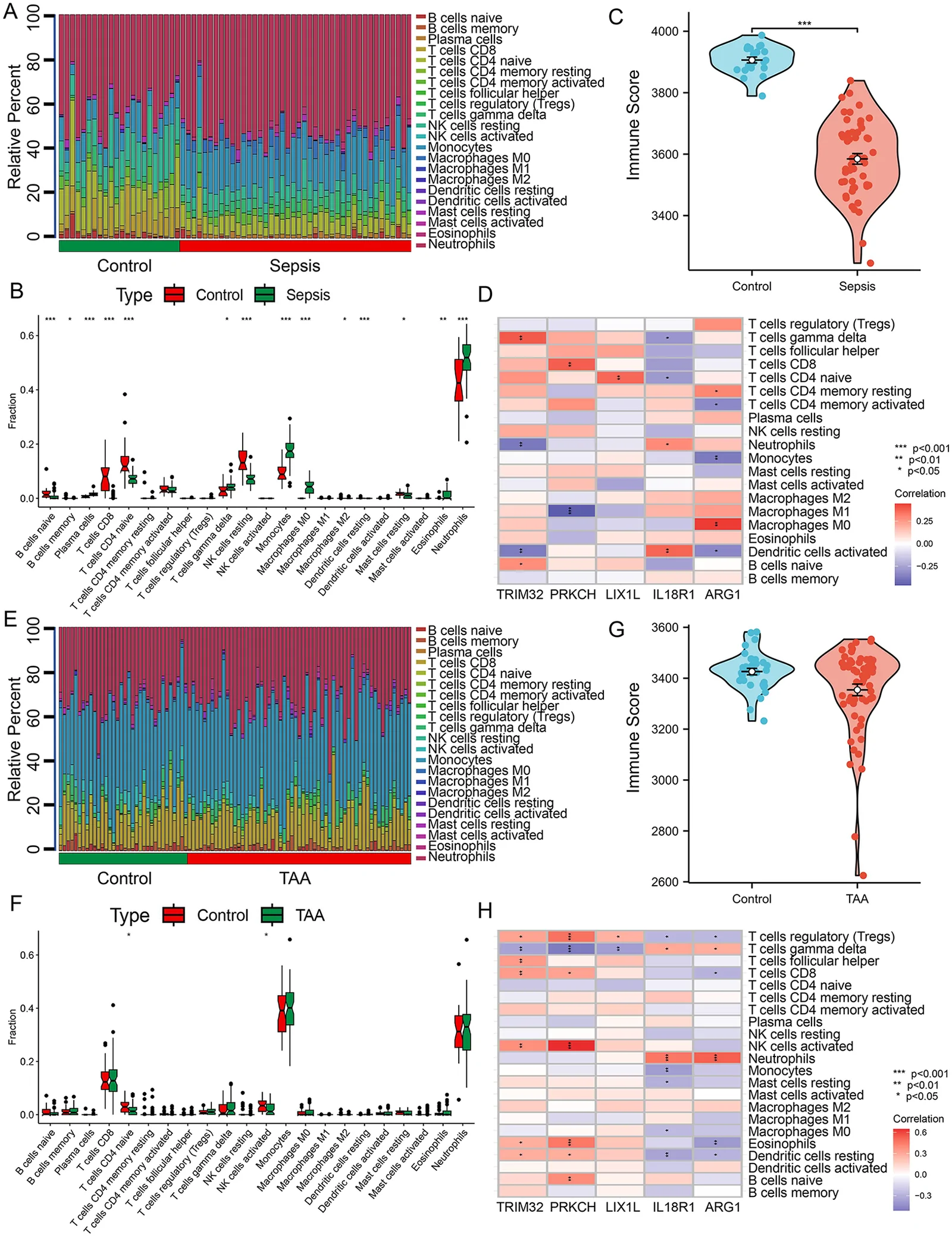
Immune cell infiltration patterns and their associations with selected genes in sepsis and TAA. A, B: Estimated proportions of 22 immune cell types in the sepsis and control groups in GSE95233. C: Comparison of immune scores between the sepsis and control groups. D: Correlations between the five selected genes and estimated immune cell proportions in sepsis. E, F: Estimated proportions of 22 immune cell types in the TAA and control groups in GSE9106. G: Comparison of immune scores between the TAA and control groups. H: Correlations between the five selected genes and estimated immune cell proportions in TAA. P < 0.05 was considered statistically significant.

In the GSE9106 dataset, two immune cell types differed significantly between the TAA and control groups (Fig 7E, F). The proportions of naive CD4 T cells and activated NK cells were lower in the TAA group than in the control group. The immune score was also lower in the TAA group, although the difference was not statistically significant (Fig 7G). Significant associations between the five selected genes and immune cell proportions were also observed in the TAA dataset (Fig 7H).

## Discussion

Sepsis and TAA are clinically distinct conditions, but both involve inflammatory dysregulation and vascular injury [29,30]. In this study, integrated transcriptomic analyses identified shared molecular features between sepsis and TAA, with prominent involvement of immune-related processes. Rather than indicating a direct causal relationship between the two conditions, these findings support the possibility that systemic inflammation in sepsis and vascular degeneration in TAA may involve partially overlapping biological responses. This molecular overlap provides a basis for further examining how inflammatory and vascular remodeling pathways may contribute to the pathophysiology of both conditions.

The immune-related signals shared between sepsis and TAA are consistent with evidence that immune dysregulation contributes to the pathophysiology of both conditions. Previous transcriptomic studies of sepsis have demonstrated marked alterations in inflammatory and adaptive immune responses, including heterogeneous T-cell-associated programs and broader changes in host immune states [31–33]. In aortic aneurysmal disease, single-cell and transcriptomic studies have likewise identified substantial immune cell involvement and interactions between inflammatory cells and vascular smooth muscle cells within the diseased aortic wall [34–36]. These observations provide biological context for the immune-related overlap identified in our analysis and suggest that altered immune regulation may represent a common feature of sepsis and TAA, although the cellular sources and consequences of these alterations are likely to differ between systemic infection and aortic disease.

The metabolic and redox-related signals observed in our analysis are also biologically plausible in both sepsis and aortic disease. In sepsis, mitochondrial dysfunction and excessive reactive oxygen species production are well-recognized features of cellular stress and can contribute to impaired endothelial and immune cell function [37]. In aortic aneurysmal disease, oxidative stress has been implicated in vascular smooth muscle cell dysfunction, extracellular matrix damage, and weakening of the aortic wall [38]. These findings from previous studies provide a biological context for the redox- and metabolism-related signatures identified in our analysis and suggest that disturbed cellular energy metabolism and oxidative stress may represent another shared biological feature of sepsis and TAA, although their relative contributions and cellular origins are likely to differ between the two conditions.

Among the five selected genes, ARG1 and IL18R1 have the strongest biological links to inflammatory regulation. Previous studies have implicated ARG1-mediated arginine metabolism in immune dysfunction and altered nitric oxide homeostasis, providing a plausible context for its association with both systemic inflammation and vascular dysfunction [39–41]. IL18R1 is a key component of IL-18 signaling, which promotes proinflammatory immune responses, including Th1-associated signaling, suggesting that IL18R1 may reflect another inflammatory pathway relevant to the molecular overlap between sepsis and TAA. In contrast, the relevance of LIX1L, TRIM32, and PRKCH to the shared biology of sepsis and TAA is less established. LIX1L has been linked to RNA-related regulatory processes [42], whereas TRIM32 and PRKCH have been implicated in inflammatory signaling and vascular cellular responses, respectively [43,44]. These previous observations provide biological plausibility for their identification in our analysis but do not establish disease-specific roles in sepsis or TAA. Thus, ARG1 and IL18R1 are supported by existing evidence related to inflammatory regulation, whereas the potential contributions of LIX1L, TRIM32, and PRKCH require further mechanistic investigation.

Several limitations should be acknowledged. First, the transcriptomic datasets were generated using different microarray platforms and biological compartments. To avoid direct cross-platform integration, each dataset was analyzed independently and shared DEGs were identified based on overlapping differential expression; nevertheless, residual platform- and tissue-related heterogeneity cannot be completely excluded. Second, the machine-learning analysis was exploratory and was developed primarily using the sepsis dataset; therefore, the selected genes should be regarded as candidate molecular features rather than established diagnostic biomarkers. Third, the experimental assessment was performed in LPS-treated rat A7R5 vascular smooth muscle cells, which represents a simplified inflammatory model and does not reproduce the complex cellular environment of human sepsis or aortic aneurysmal disease. The lack of significant changes in PRKCH and TRIM32 in this model further underscores the need for validation in human primary cells and independent clinical cohorts. Finally, GSE57691 represents abdominal rather than thoracic aortic aneurysm and was therefore used only to assess the reproducibility of selected gene expression patterns across aortic aneurysmal disease, rather than as a direct external validation cohort for TAA.

Taken together, these findings support the presence of partially shared molecular features between sepsis and TAA rather than a direct mechanistic link between the two diseases. The observed overlap should therefore be interpreted as evidence of common inflammatory and cellular stress-related signatures, without implying that the same cellular mechanisms operate in both conditions or that sepsis directly promotes TAA. Further experimental studies are required to determine whether the selected genes and pathways functionally contribute to both disease processes.

## Supporting information

S1 Table Complete list of the 193 shared differentially expressed genes (DEGs). (XLSX)

## Abbreviations

TAA: Thoracic Aortic Aneurysm
SVM: Support Vector Machine
GEO: Gene Expression Omnibus
DEGs: Differentially expressed genes
GO: Gene ontology
KEGG: Kyoto Encyclopedia of Genes and Genomes
ANN: Artificial Neural Network
GSEA: Gene set enrichment analysis
RF: Random forest
ROC: Receiver operating characteristic
AUC: Area under the curve
BP: Biological processes
CC: Cellular components
MF: Molecular function
